# The Relationship Between Genetic Variants at Loci 9p21, 6q25.1, and 2q36.3 and the Development of Cardiac Allograft Vasculopathy in Heart Transplant Patients

**DOI:** 10.3390/genes16020236

**Published:** 2025-02-19

**Authors:** Dana Dlouha, Kristyna Janouskova, Jevgenija Vymetalova, Sarka Novakova, Sarka Chytilova, Marianna Lukasova, Jaroslav A. Hubacek

**Affiliations:** 1 Center for Experimental Medicine, Institute for Clinical and Experimental Medicine, 14021 Prague, Czech Republic; jaqk@ikem.cz (K.J.); sano@ikem.cz (S.N.); jahb@ikem.cz (J.A.H.); 2 Cardio Center, Institute for Clinical and Experimental Medicine, 14021 Prague, Czech Republic; yevy@ikem.cz (J.V.); malk@ikem.cz (M.L.); 3 Statistical Unit, Institute for Clinical and Experimental Medicine, 14021 Prague, Czech Republic; sarb@ikem.cz; 4 3rd Department of Internal Medicine, 1st Faculty of Medicine, Charles University, 11636 Prague, Czech Republic

**Keywords:** SNPs, transplantation, cardiac allograft vasculopathy

## Abstract

Background: Cardiac allograft vasculopathy (CAV) is an accelerated form of coronary artery disease (CAD) that is characterized by concentric fibrous intimal hyperplasia along the length of coronary vessels, and is recognized as long-term complication after heart transplantation. The chromosomal loci 9p21, 6q25.1, and 2q36.3, represented by their respective leading variants rs10757274, rs6922269 and rs2943634, have been linked with a history of CAD by genome-wide association studies. We aimed to investigate the associations of genetic variants at the loci 9p21, 6q25.1, and 2q36.3 with CAV as genetic risk factors for early prediction. Methods: Genomic DNA was extracted from paired aortic samples of 727 heart recipients (average age 50.8 ± 12.2 years; 21.3% women) and corresponding donors (average age 39.7 ± 12.0 years; 26.1% women). The variants within the loci 9p21, 6q25.1, and 2q36.3 were genotyped using PCR-RFLP. Results: The recipients’ variants of 9p21 (OR 1.97; 95% CI, 1.21-3.19 for GG vs. +A comparison, *p* = 0.0056) and 2q36.3 (OR 2.46; 95% CI, 1.12–6.17 for +C vs. AA comparison, *p* = 0.0186) were associated with higher incidence of CAV during the first year following heart transplantation. No such association was found for donor genotypes. Conclusions: Our data suggest that variants at the locus 9p21 (rs10757274) and 2q36.3 (rs2943634) are associated with early CAV development.

## 1. Introduction

Cardiac allograft vasculopathy (CAV) remains the leading long-term cause of death and re-transplantation following heart transplantation (HTx). According the data from the most recent International Society of Heart and Lung Transplantation (ISHLT) registry, the prevalence of CAV is reported to be 7.7% in the first year following adult heart transplantation, 29% in the next 5 years, and 46.8% in the following 10 years [1]. The development of CAV is a multifactorial and complex process, initiated by heterogeneous factors that ultimately cause inflammation and endothelial injury [2]. CAV is generally considered to be primarily an immunologically mediated disease. The two major immune risk factors associated with the development of CAV are the presence of alloantibody and occurrence of acute rejection [3,4], but the real genesis of CAV remains elusive.

Identification of genes involved in the process of CAV may provide a novel insight into pathophysiology and lead to new therapeutic options. Genetic traits in cytokine, growth factor genes, and β-adrenergic receptors have previously been considered important risk factors for cardiac transplant-related CAV development [5,6,7,8,9,10,11]. Except for one study [10], all existing research has involved only a few dozen subjects, which may have affected the reliability of the results.

Genome-wide association studies (GWAS) have revealed that chromosomal loci 9p21, 6q25.1, and 2q36.3, represented by their respective leading single nucleotide polymorphisms (SNPs) rs10757274, rs6922269 and rs2943634, have been linked with a history of coronary artery disease (CAD) [12,13]. The locus 9p21 (rs10757274) is located within *ANRIL* (antisense non-coding RNA in the *INK4* locus; OMIM acc. N. 613149) [14]. *ANRIL* is clustered within the cyclin-dependent kinase inhibitors, CDKN2A and CDKN2B. It is known to regulate various processes related to endothelial injury and the subsequent development of atherosclerosis through a complex mechanism [15]. The fact that *ANRIL* is expressed in nearly all human tissues underscores the significant importance of this regulatory RNA [16]. The locus 6q25.1 (rs6922269), an intron variant in the *MTHFD1L* gene (methylenetetrahydrofolate dehydrogenase 1-like, NADP (+)-dependent; this enzyme is involved in cellular methylation reactions through the regeneration of methionine from homocysteine; OMIM acc. No 611427). The functional effect of rs6922269 is currently unknown [17,18]. The locus 2q36.3 (rs2943634) is located within an intergenic region 500 kb from the gene encoding insulin receptor substrate-1 (*IRS1* locus), and has been associated with CAD [12,19]. The potential mechanism leading to the increased cardiovascular disease risk is not known in detail for this locus, as the variant is located within the “gene-free” zone [17].

CAV is an accelerated form of CAD that is characterized by concentric fibrous intimal hyperplasia along the length of coronary vessels [20,21]. There are numerous similarities and differences of CAV with focal atherosclerotic lesions of proximal coronary arteries in native hearts. Both conditions are marked by increased expression of cell adhesion molecules, leukocyte infiltration, similar cytokine profiles, and an abnormal accumulation of extracellular matrix. Additionally, both diseases exhibit early and prolonged accumulation of extracellular and intracellular lipids. Intimal smooth muscle cell migration, endothelial dysfunction, and abnormal apoptosis are also observed in both conditions [22]. We investigated the associations of rs10757274, rs6922269 and rs2943634 with CAV as genetic risk factors for early prediction.

## 2. Materials and Methods

### 2.1. Study Subjects

The study was performed according to the Declaration of Helsinki (2000) of the World Medical Association. All patients signed informed consent forms, confirming their participation in the study. The consent form states that a small amount (approximately 200 mg) of aortic tissue will be collected during transplantation to obtain genetic material for gDNA isolation and examination of genetic polymorphisms. Additionally, the consent form includes permission for the anonymous use of patients’ DNA for medical research purposes. It also specifies that the results of the testing, including information about patients’ health status related to this research, may be presented in professional scientific circles or published in journals without any identifying information or attribution. The basic characteristics of the patients are shown in Table 1. Samples of patients who underwent HTx from January 2005 to October 2023 at the Cardio Center of the Institute for Clinical and Experimental Medicine, Prague, Czech Republic were included in the study. Paired aortic samples of 727 heart recipients (average age 50.8 ± 12.2 years; 21.3% women) and corresponding donors (average age 39.7 ± 12.0 years; 26.1% women) were taken during HTx. Patients were subjected to a standard post-transplant clinical follow-up program. Histology, immunohistochemistry, and endomyocardial biopsy (EMB) were performed according to standard protocols and guidelines [23,24,25]. Coronary angiography was conducted one year after transplantation as a screening and surveillance test for CAV. Corticosteroids and tacrolimus were administered as initial immunosuppressive therapy in all subjects. Biochemical parameters were measured as a part of routine standard practice.

Data are presented as counts (%), mean ± standard deviation (SD), median, and interquartile range (IQR).

### 2.2. Genotyping

**Figure 1:**
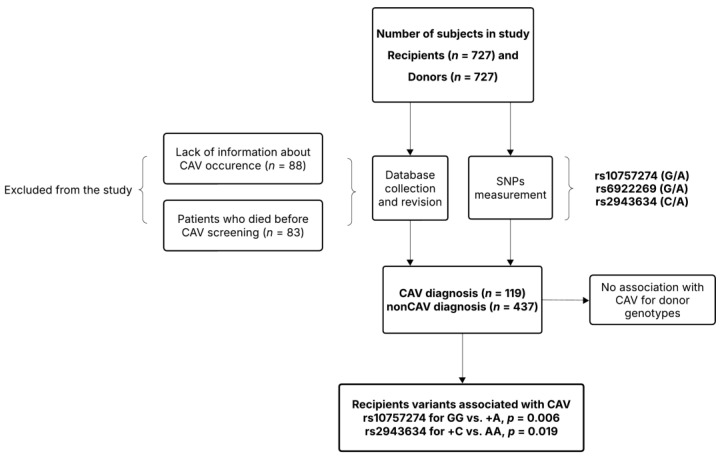
Flow diagram of the study.

The flow diagram of the study is presented in Figure 1. Genomic DNA was extracted from 100 mg of aortic tissue using a standard salting-out method [26]. All variants were examined via polymerase chain reaction (PCR) and restriction fragment analysis. DNA amplifications were carried out in a 25 μL reaction volume, which included 1 μL of DNA template, 1 μL of each relevant forward and reverse primer (10 μM; Metabion International AG, Planegg, Germany), 0.5 μL of dNTP (100 mM; Thermo Fisher Scientific, Waltham, MA, USA), 1.5 μL of MgCl_2_ (25 mM; Thermo Fisher Scientific, MA, USA), 2.5 μL of 10× DreamTaq Buffer (which contains 20 mM MgCl_2_; Thermo Fisher Scientific, MA, USA), 0.05 μL of Dream Taq DNA Polymerase (5 U/μL; Thermo Fisher Scientific, Waltham, MA, USA), and 17.45 μL of nuclease-free water (MERCK, Burlington, MA, USA). The cycling profile for each variant began with polymerase activation at 95 °C for 3 min, followed by DNA denaturation at 95 °C for 15 s. Annealing occurred at a temperature specific to the SNPs for 30 s, followed by DNA extension at 72 °C. The steps from DNA denaturation to extension were repeated 34 times, after which a final extension occurred at 72 °C for 3 min, with the reaction held at 4 °C indefinitely. PCR was performed using a Bio-Rad™ Thermal Cycler (Bio-Rad Laboratories, Hercules, CA, USA). Detailed primer sequences, annealing temperature, and restriction enzymes are shown in Supplementary Material Table S1.

### 2.3. Statistical Analyses

The statistical analysis was conducted using R version 4.4.1. To evaluate differences in the abundance of various alleles across three SNPs between recipients and donors, a Poisson regression model was employed. The dependent variable was the number of patients, with recipient–donor group membership and genotype included as covariates. For the analysis of CAV in relation to different genotypes, a negative binomial regression model was used, allowing for dispersion to be modeled according to CAV status and genotype. The dependent variable was the number of patients, and the covariates included genotype, CAV (binary), recipient age, recipient sex, and primary etiology of heart failure. Each SNP was analyzed in a separate model. Marginal means were estimated using least squares means derived from the means package (version 1.10.5). To analyze the genetic predisposition for CAV, the unweighted genetic risk score (GRS) was created according to the simple cumulative presence of different alleles—2 points for risk (CAV occurrence) allele homozygotes, 1 point for heterozygotes, and 0 points for subjects without any risk allele. The association between CAV and the cumulative risk allele score was evaluated using both the negative binomial regression model and the Cochran–Armitage trend test. GRS was calculated for both recipient and donor genotypes. *p* values less than 0.05 were considered significant.

## 3. Results

### 3.1. Main Characteristics

Allograft recipients were older than their donors (*p* < 0.0001), and the sex ratio was similar in both groups. The dominant primary heart diseases diagnosed among the patients were non-ischemic dilated cardiomyopathy (*N* = 342) and ischemic dilated cardiomyopathy (*N* = 254). A left ventricular assist device (LVAD) was implanted in 32.6% of the patients as a bridge to transplantation. The median duration of LVAD support was 342 days, ranging from 7 to 2509 days. The most common post-transplant complication was acute rejection, with the majority being of the acute cellular rejection subtype (*N* = 273). Antibody-mediated rejection occurred in 84 subjects. CAV was diagnosed in 119 patients within the first year following HTx. From the study cohort, 257 patients had already died by the time of analysis. The median survival time was 3.8 years, with an interquartile range of 0.3 to 8.3 years.

### 3.2. Genotypes of Recipients and Donors

Similar genotype frequencies for all SNPs were identified in recipients and donors (Table 2). When both groups were analyzed, all SNPs were found to be in Hardy–Weinberg equilibrium. When recipients and donors were tested for Hardy–Weinberg equilibrium separately, all SNPs were in Hardy–Weinberg equilibrium, except for the variant at the 9p21 locus (rs10757274) in donors.

Genotype frequencies differed between patients who experienced CAV and those who did not within the loci 9p21 (*p* = 0.012) and 2q36.3 (*p* = 0.06; Table 3) and remained different after adjustment for covariates (age, sex, and primary heart disease). Variants of 9p21 (OR 1.97; 95% CI, 1.21–3.19 for GG vs. +A comparison, *p* = 0.0056) and 2q36.3 (OR 2.46; 95% CI, 1.12–6.17 for +C vs. AA comparison, *p* = 0.0186) were associated with higher incidence of CAV (Table 4). After adjustment for covariates (, this association remained significant (for both *p* adj ≤ 0.026). No such association was found for donor genotypes.

**Figure 2:**
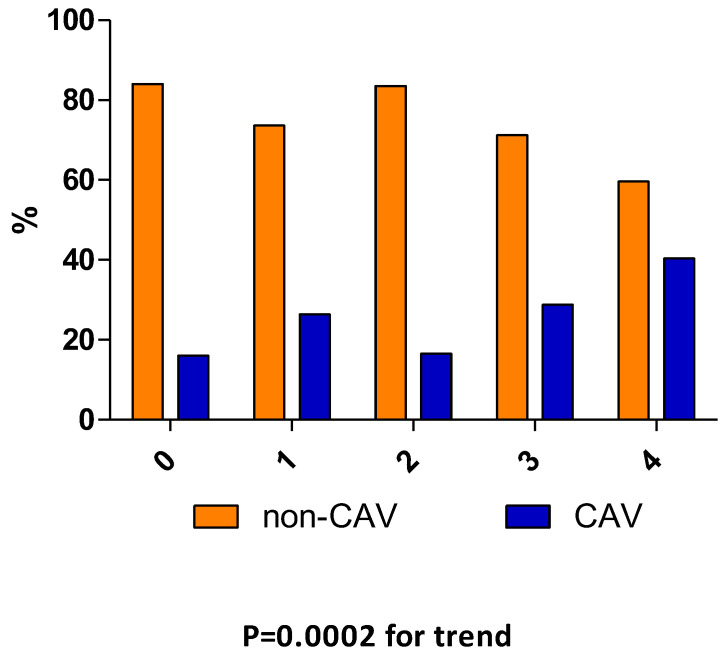
Distribution of GRS 2+9 in CAV vs. non-CAV recipients. The data represent a percentage of the total risk alleles found in 2q36.3 (rs2943634) and 9p21 (rs10757274). The incidence of CAV displayed a linear trend: 16% of subjects had a risk score of 0, compared to 40% of subjects with a score of 4.

Additionally, GRS was created by pooling risk alleles of all measured SNPs (GRS all) and for the 2q36.3 and 9p21 (GRS 2+9) specifically in recipients. GRS analyses revealed a connection between the cumulative presence of risky alleles within loci 9p21 and 2q36.3 and CAV only (*p* adj = 0.012; Table 5). In GRS 2-9, the incidence of CAV displayed a linear trend: 16% of subjects had a risk score of 0, compared to 40% of subjects with a score of 4 (*p* = 0.0002 for trend, Figure 2).

## 4. Discussion

Despite ongoing efforts in prevention and treatment, a significant number of HTx patients continue to experience notable progression of CAV, particularly within the first year following the transplant procedure. The aim of our study was to find a new genetic risk marker for early detection of patients at a risk of developing CAV. We identified the recipients’ variants GG at the locus 9p21, and +C at the locus 2q36.3 to be associated with higher incidence of CAV. Moreover, an association between the cumulative presence of risky alleles at both loci and the occurrence of CAV was identified. Our study is the first that has focused on the SNPs that have been previously described in association with CAD. Recent reports indicate that genes expressed in the 9p21.3 locus play a crucial role in regulating various essential functions within vascular cells, including cell proliferation and inflammatory responses. Additionally, these genes have systemic metabolic effects that are relevant to cardiovascular diseases (CVDs) [27]. Furthermore, genetic variants located in the 9p21.3 locus have been associated with increased cellular senescence in both vascular smooth muscle cells and endothelial cells. This senescence leads to diminished cellular repair and regeneration capacities [28,29]. CAV is characterized by injury and inflammation of the coronary endothelium, involving smooth muscle proliferation, lipid deposition, and the accumulation of inflammatory cells [3]. The GG variant of rs10757274 likely contributes to *ANRIL*’s dysfunction, leading to increased endothelial injury during the progression of CAV. The contribution of locus 2q36.3, that has previously been strongly associated with CAD, may also suggest the influence of genes expressed within this locus, which are associated with insulin resistance, type 2 diabetes mellitus (T2DM), triglycerides, and HDL-cholesterol concentrations [19]. We did not find a connection between the occurrence of T2DM and the risk of CAV in our study. A previous study has identified that rs2943634 is associated with plasma levels of HDL-cholesterol and adiponectin [30]. The exact mechanism by which rs2943634 contributes to the development of CAV is unclear, but it may affect lipid metabolism and deposition.

The current guideline-recommended diagnostic modality for invasive CAV screening is coronary angiography. Newer diagnostic methodologies include the use of precision medicine, optical coherence tomography, coronary computed tomography angiography with fractional flow reserve, and cardiac magnetic resonance imaging. [20,31]. Using the screening of candidate SNPs may offer the ability to identify at-risk patients before or soon after HTx; this will allow these patients to be closely monitored and screened in an effort to prevent potential complications or death related to CAV.

The limitation of our retrospective study is the absence of information on the occurrence of CAV in eighty-eight patients who were transplanted between 2005 and 2009, as well as in eighty-three patients who died before CAV screening during the first year post-HTx.

## 5. Conclusions

Our data suggest that variants at the loci 9p21 (rs10757274) and 2q36.3 (rs2943634) are associated with early CAV development.

## Figures and Tables

**Table 1:** Demographics of the patients.

Characteristics	Graft Recipients	Graft Donors	*p* Value
N (females %)	727 (21.3%)	727 (26.1%)	n.s.
Age, years	50.8 ± 12.2	39.7 ± 12.0	<0.0001
Diabetes mellitus	162 (22.3%)		
RI	192 (28.7%)		
Etiology of non-ischemic DCM	342 (47%)		
Etiology of ischemic DCM	254 (35.1%)		
Congenital heart defects	45 (6.2%)		
Others	86 (11.7%)		
LVAD implant before HTx	236 (32.6%)		
Days of LVAD support (days)	342 (425)		
CAV within 1st year post HTx	119 (16.4%)		
ACR	273 (39.7%)		
AMR	84 (12.2%)		
Death	257 (35.4%)		
Survival time	3.8 (8)		

ACR, acute cellular rejection; AMR, antibody-mediated rejection; CAV, cardiac allograft vasculopathy; DCM, dilated cardiomyopathy; LVAD, left ventricular assist device; RI, renal insufficiency.

**Table 2:** Genotype frequencies of analyzed polymorphisms within the groups of recipients and donors. *p* values were calculated by Chi-square tests.

SNP	Genotype	Recipients		Donors		*p* Value
		*N*	%	*N*	%	
2q36.3 rs2943634	AA	86	12.1	83	11.9	0.40
	CA	325	45.8	295	42.4	
	CC	299	42.1	317	45.6	
6q25.1 rs6922269	AA	50	6.9	53	7.6	0.89
	GA	287	39.9	275	39.3	
	GG	383	53.2	372	53. 1	
9p21 rs10757274	AA	191	26.7	214	30.6	0.12
	AG	363	50.8	318	45.5	
	GG	161	22.5	167	23.9	

**Table 3:** Comparison of recipient and donor SNPs distribution between patients with CAV and without CAV. * *p* values adjusted for age, sex, and primary heart disease.

SNP	Genotype	Recipients						Donors					
		non-CAV		CAV				non-CAV		CAV			
		*N*	%	*N*	%	*p* Value	* *p* Value	*N*	%	*N*	%	*p* Value	* *p* Value
2q36.3 rs2943634	AA	57	15.4	8	6.9	**0.06**	**0.05**	46	12.8	13	11.7	0.61	0.8
	CA	166	45.0	56	48.3			149	41.5	52	46.8		
	CC	146	39.6	52	44.8			164	45.7	46	41.4		
6q25.1 rs6922269	AA	23	6.1	11	9.3	0.42	0.6	31	8.6	5	4.4	0.28	0.6
	GA	147	39.2	42	35.6			147	40.6	44	38.9		
	GG	205	54.7	65	55.1			184	50.8	64	56.6		
9p21 rs10757274	AA	105	28.2	30	25.9	**0.012**	**0.04**	108	30.1	38	33.6	0.73	0.7
	AG	191	51.3	47	40.5			166	46.2	48	42.5		
	GG	76	20.4	39	33.6			85	23.7	27	23.9		

**Table 4:** The recipients’ genetic risk for CAV occurrence for individual variants. * *p* values adjusted for age, sex, and primary heart disease.

SNP	Genotype	OR	95%CI	*p* Value		OR	95%CI	*p* Value	* *p* Value
2q36.3 rs2943634	AA	1.00			AA	1.00			
	CA	2.36	1.06, 5.25	**0.04**	CC + CA	2.46	1.12, 6.18	**0.019**	**0.021**
	CC	2.59	1.16, 5.78	**0.017**					
6q25.1 rs6922269	AA	1.00							
	GA	0.59	0.26, 1.31	0.19					
	GG	0.67	0.31, 1.45	0.3					
9p21 rs10757274	AA	1.00			AA + AG	1.00			
	AG	0.88	0.53, 1.48	0.69					
	GG	1.79	1.02, 3.13	**0.047**	GG	1.97	1.21, 3.19	**0.006**	**0.026**

**Table 5:** The relationship between GRS and the occurrence of CAV. GRS represents the sum of the risk alleles of all measured SNPs (GRS all), and the sum of the risk alleles from the loci 2q36.3 and 9p21 (GRS 2+9). 0—without CAV; 1—with CAV; * *p* values adjusted for age, sex, and primary heart disease.

GRS All	CAV						GRS 2+9	CAV					
	0		1					0		1			
	*N*	%	*N*	%	*p* Value	* p Value		*N*	%	*N*	%	*p* Value	* p Value
0	1	0.3	1	0.8	0.25	0.3	0	21	5.6	4	3.4	**0.0026**	**0.012**
1	15	4.0	3	2.5			1	66	17.5	24	20.2		
2	38	10.1	9	7.6			2	166	44.0	33	27.7		
3	116	30.8	34	28.6			3	93	24.7	37	31.1		
4	130	34.5	35	29.4			4	31	8.2	21	17.6		
5	63	16.7	28	23.5									
6	14	3.7	9	7.6									

## Data Availability

The original contributions presented in the study are included in the article/Supplementary Material, further inquiries can be directed to the corresponding author.

## Supplementary Materials

The following supporting information can be downloaded at: https://www.mdpi.com/article/10.3390/genes16020236/s1 [26], Table S1: Primer sequences and PCR conditions; Figure S1: Locus 9p21 (rs10757274); Figure S2: Locus 6q25.1 (rs6922269); Figure S3: Locus 2q36.3 (rs2943634).

## References

[1] Khush K.K., Cherikh W.S., Chambers D.C., Harhay M.O., Hayes D., Hsich E., Meiser B., Potena L., Robinson A., Rossano J.W. (2019). International Society for Heart and Lung Transplantation. The International Thoracic Organ Transplant Registry of the International Society for Heart and Lung Transplantation: Thirty-sixth adult heart transplantation report—2019; focus theme: Donor and recipient size match. J. Heart Lung Transplant..

[2] Michieli L., Lin C., Tona F. (2019). Non-Invasive Assessment of Coronary Microcirculation in Heart Transplantation. World J. Cardiovasc. Dis..

[3] Lee F., Nair V., Chih S. (2020). Cardiac allograft vasculopathy: Insights on pathogenesis and therapy. Clin. Transplant..

[4] Costa D., Picascia A., Grimaldi V., Amarelli C., Petraio A., Levi A., Di Donato M., Pirozzi A.V.A., Fiorito C., Moccia G. (2024). Role of HLA matching and donor specific antibody development in long-term survival, acute rejection and cardiac allograft vasculopathy. Transpl. Immunol..

[5] Densem C.G., Hutchinson I.V., Cooper A., Yonan N., Brooks N.H. (2000). Polymorphism of the transforming growth factor-beta 1 gene correlates with the development of coronary vasculopathy following cardiac transplantation. J. Heart Lung Transplant..

[6] Densem C.G., Hutchinson I.V., Yonan N., Brooks N.H. (2003). Influence of interleukin-10 polymorphism on the development of coronary vasculopathy following cardiac transplantation. Transpl. Immunol..

[7] Densem C.G., Ray M., Hutchinson I.V., Yonan N., Brooks N.H. (2005). Interleukin-6 polymorphism: A genetic risk factor for cardiac transplant related coronary vasculopathy?. J. Heart Lung Transplant..

[8] Ternstrom L., Jeppsson A., Ricksten A., Nilsson F. (2005). Tumor necrosis factor gene polymorphism and cardiac allograft vasculopathy. J. Heart Lung Transplant..

[9] Tambur A.R., Pamboukian S., Costanzo M.R., Heroux A. (2006). Genetic polymorphism in platelet-derived growth factor and vascular endothelial growth factor are significantly associated with cardiac allograft vasculopathy. J. Heart Lung Transplant..

[10] Khush K.K., Pawlikowska L., Menza R.L., Goldstein B.A., Hayden V., Nguyen J., Kim H., Poon A., Sapru A., Matthay M.A. (2012). Beta-adrenergic receptor polymorphisms and cardiac graft function in potential organ donors. Am. J. Transplant..

[11] Mayerova L., Chaloupka A., Wohlfahrt P., Hubacek J.A., Bedanova H., Chen Z., Kautzner J., Melenovsky V., Malek I., Tomasek A. (2023). Role of genetics in the development of cardiac allograft vasculopathy. Bratisl. Lek. Listy..

[12] Samani N.J., Erdmann J., Hall A.S., Hengstenberg C., Mangino M., Mayer B., Dixon R.J., Meitinger T., Braund P., Wichmann H.E. (2007). WTCCC and the Cardiogenics Consortium. Genomewide association analysis of coronary artery disease. N. Engl. J. Med..

[13] Muendlein A., Saely C.H., Rhomberg S., Sonderegger G., Loacker S., Rein P., Beer S., Vonbank A., Winder T., Drexel H. (2009). Evaluation of the association of genetic variants on the chromosomal loci 9p21.3, 6q25.1, and 2q36.3 with angiographically characterized coronary artery disease. Atherosclerosis.

[14] Helgadottir A., Thorleifsson G., Manolescu A., Gretarsdottir S., Blondal T., Jonasdottir A., Jonasdottir A., Sigurdsson A., Baker A., Palsson A. (2007). A common variant on chromosome 9p21 affects the risk of myocardial infarction. Science.

[15] Cho H., Shen G.Q., Wang X., Wang F., Archacki S., Li Y., Yu G., Chakrabarti S., Chen Q., Wang Q.K. (2019). Long noncoding RNA *ANRIL* regulates endothelial cell activities associated with coronary artery disease by up-regulating *CLIP1*, *EZR*, and *LYVE1* genes. J. Biol. Chem..

[16] Zhang Y.N., Qiang B., Fu L.J. (2020). Association of ANRIL polymorphisms with coronary artery disease: A systemic meta-analysis. Medicine.

[17] Hubacek J.A., Staněk V., Gebauerová M., Poledne R., Aschermann M., Skalická H., Matoušková J., Kruger A., Pěnička M., Hrabáková H. (2015). Rs6922269 marker at the MTHFD1L gene predict cardiovascular mortality in males after acute coronary syndrome. Mol. Biol. Rep..

[18] Palmer B.R., Slow S., Ellis K.L., Pilbrow A.P., Skelton L., Frampton C.M., Palmer S.C., Troughton R.W., Yandle T.G., Doughty R.N. (2014). Genetic polymorphism rs6922269 in the MTHFD1L gene is associated with survival and baseline active vitamin B12 levels in post-acute coronary syndromes patients. PLoS ONE.

[19] Lim S., Hong J., Liu C.T., Hivert M.F., White C.C., Murabito J.M., O’Donnell C.J., Dupuis J., Florez J.C., Meigs J.B. (2013). Common variants in and near IRS1 and subclinical cardiovascular disease in the Framingham Heart Study. Atherosclerosis.

[20] Pober J.S., Chih S., Kobashigawa J., Madsen J.C., Tellides G. (2021). Cardiac allograft vasculopathy: Current review and future research directions. Cardiovasc. Res..

[21] Ramzy D., Rao V., Brahm J., Miriuka S., Delgado D., Ross H.J. (2005). Cardiac allograft vasculopathy: A review. Can. J. Surg..

[22] Rahmani M., Cruz R.P., Granville D.J., McManus B.M. (2006). Allograft vasculopathy versus atherosclerosis. Circ. Res..

[23] Berry G.J., Burke M.M., Andersen C., Bruneval P., Fedrigo M., Fishbein M.C., Goddard M., Hammond E.H., Leone O., Marboe C. (2013). The 2013 International Society for Heart and Lung Transplantation Working Formulation for the standardization of nomenclature in the pathologic diagnosis of antibody-mediated rejection in heart transplantation. J. Heart Lung Transpl..

[24] Stewart S., Winters G.L., Fishbein M.C., Tazelaar H.D., Kobashigawa J., Abrams J., Andersen C.B., Angelini A., Berry G.J., Burke M.M. (2005). Revision of the 1990 working formulation for the standardization of nomenclature in the diagnosis of heart rejection. J. Heart Lung Transpl..

[25] Cooper L.T., Baughman K.L., Feldman A.M., Frustaci A., Jessup M., Kuhl U., Levine G.N., American Heart Association, American College of Cardiology, European Society of Cardiology (2007). The role of endomyocardial biopsy in the management of cardiovascular disease: A scientific statement from the American Heart Association, the American College of Cardiology, and the European Society of Cardiology. Circulation.

[26] Miller S.A., Dykes D.D., Polesky H.F. (1988). A simple salting out procedure for extracting DNA from human nucleated cells. Nucleic Acids Res..

[27] Holdt L.M., Teupser D. (2018). Long noncoding RNA ANRIL: Lnc-ing genetic variation at the chromosome 9p21 locus to molecular mechanisms of atherosclerosis. Front. Cardiovasc. Med..

[28] Gorenne I., Kavurma M., Scott S., Bennett M. (2006). Vascular smooth muscle cell senescence in atherosclerosis. Cardiovasc. Res..

[29] Minamino T., Miyauchi H., Yoshida T., Ishida Y., Yoshida H., Komuro I. (2002). Endothelial cell senescence in human atherosclerosis: Role of telomere in endothelial dysfunction. Circulation.

[30] Arregui M., Fisher E., Knüppel S., Buijsse B., di Giuseppe R., Fritsche A., Corella D., Willich S.N., Boeing H., Weikert C. (2012). Significant associations of the rs2943634 (2q36.3) genetic polymorphism with adiponectin, high density lipoprotein cholesterol and ischemic stroke. Gene.

[31] Shahandeh N., Kashiyama K., Honda Y., Nsair A., Ali Z.A., Tobis J.M., Fearon W.F., Parikh R.V. (2022). Invasive Coronary Imaging Assessment for Cardiac Allograft Vasculopathy: State-of-the-Art Review. J. Soc. Cardiovasc. Angiogr. Interv..

[32] Janouskova K., Hubacek J.A., Vymetalova J., Novakova S., Chytilova S., Lukasova M., Dlouha D. The association of genetic variants on the chromosomal loci 9p21, 6q25.1, and 2q36.3 with cardiac allograft vasculopathy development in patients after heart transplantation. Proceedings of the 28th Congress on Atherosclerosis.

