# Supplementary material for: The Relationship Between Genetic Variants at Loci 9p21, 6q25.1, and 2q36.3 and the Development of Cardiac Allograft Vasculopathy in Heart Transplant Patients
Source: Genes (Basel). 2025 Feb 19;16(2):236. doi: 10.3390/genes16020236 (PMC11855879; doi:10.3390/genes16020236)
Supplement: Supplementary file 1 [file genes-16-00236-s001.zip › genes-3477988-supplementary.pdf]

Genotyping protocol

Genomic DNA was extracted from 100 mg of aortic tissue using the standard salting-out method described by Miller et al. (1988). DNA amplifications were carried out in a 25 µl reaction volume, which included 1 µl of DNA template, 1 µl of each relevant forward and reverse primer (10 µM; Metabion, Germany), 0.5 µl of dNTP (100 mM; ThermoFisher Scientific, USA), 1.5 µl of MgCl2 (25 mM; ThermoFisher Scientific, USA), 2.5 µl of 10x DreamTaq Buffer (which contains 20 mM $MgCl_2$; ThermoFisher Scientific, USA), 0.05 µl of Dream Taq DNA Polymerase (5 U/µl; ThermoFisher Scientific, USA), and 17.45 µl of nuclease-free water (Sigma, United Kingdom). The cycling profile for each variant began with polymerase activation at 95°C for 3 minutes, followed by DNA denaturation at 95°C for 15 seconds. Annealing occurred at a temperature specific to the SNPs for 30 seconds, followed by DNA extension at 72°C. Steps from DNA denaturation to extension were repeated 34 times, after which a final extension occurred at 72°C for 3 minutes, with the reaction held at 4°C indefinitely. Polymerase chain reaction (PCR) was performed using a Bio-Rad™ Thermal Cycler (Bio-Rad Laboratories, USA). Following this, PCR products were electrophoresed in a 3% agarose gel stained with ethidium bromide.

Detailed primer sequences, PCR conditions including the annealing temperature, and relevant restriction enzymes can be found in Supplementary Material S Table 1.

The PCR products were digested with appropriate restriction enzymes at 37°C overnight. Restriction reactions were performed in a total volume of 20 µl. The reaction mixture included 10 µl of PCR product, 0.2 µl of 10x Buffer Tango (containing BSA, ThermoFisher Scientific, USA) for variants rs10757274 and rs2943634, or 0.2 µl of 10x Buffer G (containing BSA, ThermoFisher Scientific, USA) for variant rs6922269. Additionally, 0.1 µl of the appropriate restriction enzyme (10 U/µl, ThermoFisher Scientific, USA) was used for rs6922269 and

rs2943634, while 0.3 µl was used for rs10757274. Finally, 7.7 to 7.9 µl of nuclease-free water (Sigma, United Kingdom) was added to complete the reaction.

The restriction fragments were electrophoresed in a 10% acrylamide gel, also stained with ethidium bromide.

Table S1. Primer sequences and PCR conditions

Variant	Primer Sequence		PCR		RFLP	
	Forward	Reverse	Annealing temperature	PCR product size	Restriction Enzyme	Restriction fragments
9p21 (rs10757274)	5'-TTG CTT GGT AGA TCT TCC TCC ATC CCT T-3'	5'-TTC CCA GAT GCA CTG TAT TGT TTG CCT TAC-3'	66°C	225 bp	BsmaI (Alw26I)	125 + 100 bp
6q25.1 (rs6922269)	5'-TTT ACC ACT TCT CTC TGG ATA AGC C-3'	5'-AAT GAT GTC TTT TTA AAG ATG AGT AAT A-3'	50°C	149 bp	SspI	121 + 28 bp
2q36.3 (rs2943634)	5'-AAA GCA AGC ACA TCT GTG GCT GTA C-3'	5'-TAC ACT TGA AAA TTG TAG TTG CTC C-3'	57.4°C	150 bp	Bsp1407I (BsrGI)	124 + 26 bp

Gel images show the restricted fragments of all analyzed variants. The gels were run on a 3% agarose gel stained with ethidium bromide. The GeneRuler 100 bp DNA Ladder from ThermoFisher Scientific served as the size standard.

Figure S1 Locus 9p21 (rs10757274)

The first lane contains the ladder, the second lane shows the homozygous GG variant (225 bp), the third lane represents the heterozygous AG variant (225/ 125 bp), the fourth lane depicts the homozygous AA variant (125 bp + 100 bp), and the fifth lane displays the non-restricted PCR product (225 bp).

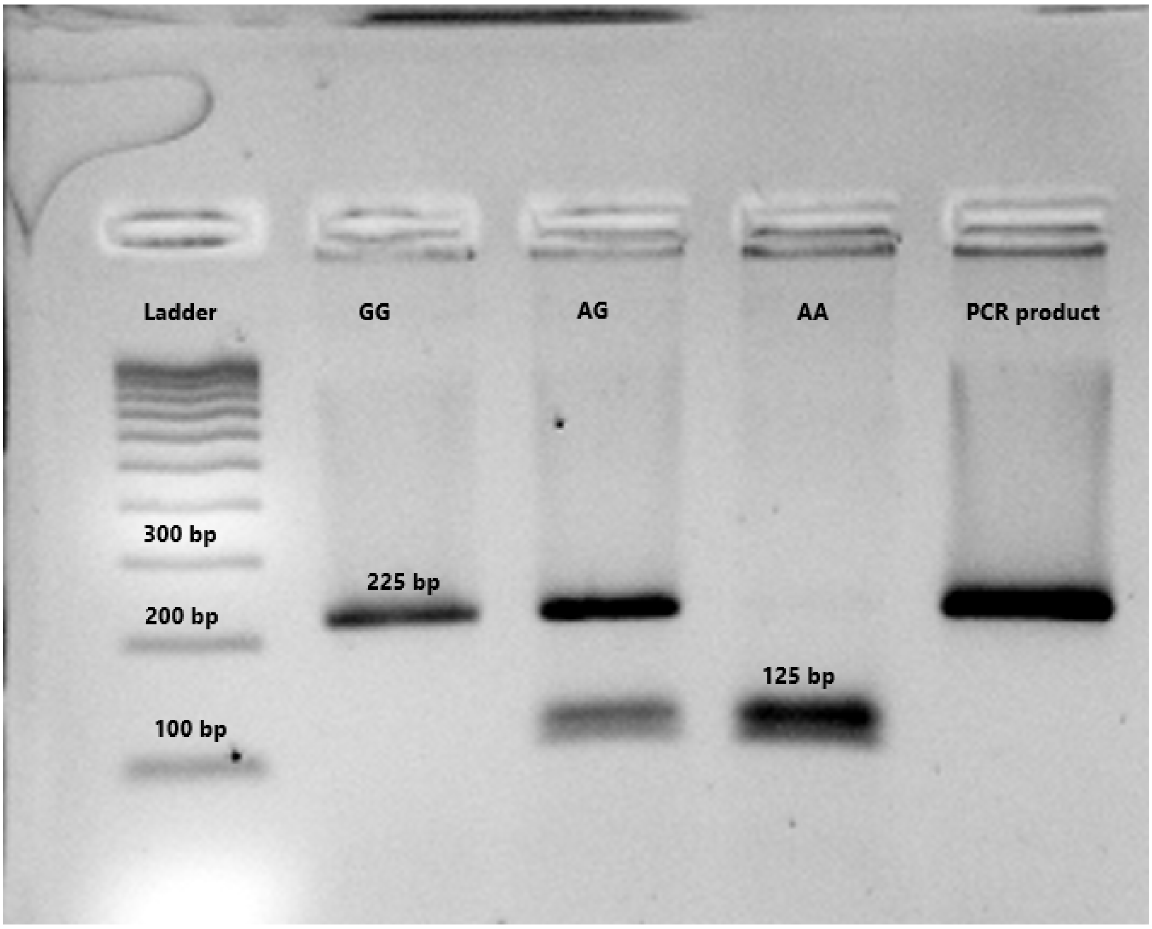

Figure S2 Locus 6q25.1 (rs6922269)

The first lane contains the ladder, the second lane shows the homozygous GG variant (149 bp), the third lane represents the heterozygous AG variant (149/ 121 bp), the fourth lane depicts the homozygous AA variant (121 bp + 28 bp), and the fifth lane displays the non-restricted PCR product (149 bp).

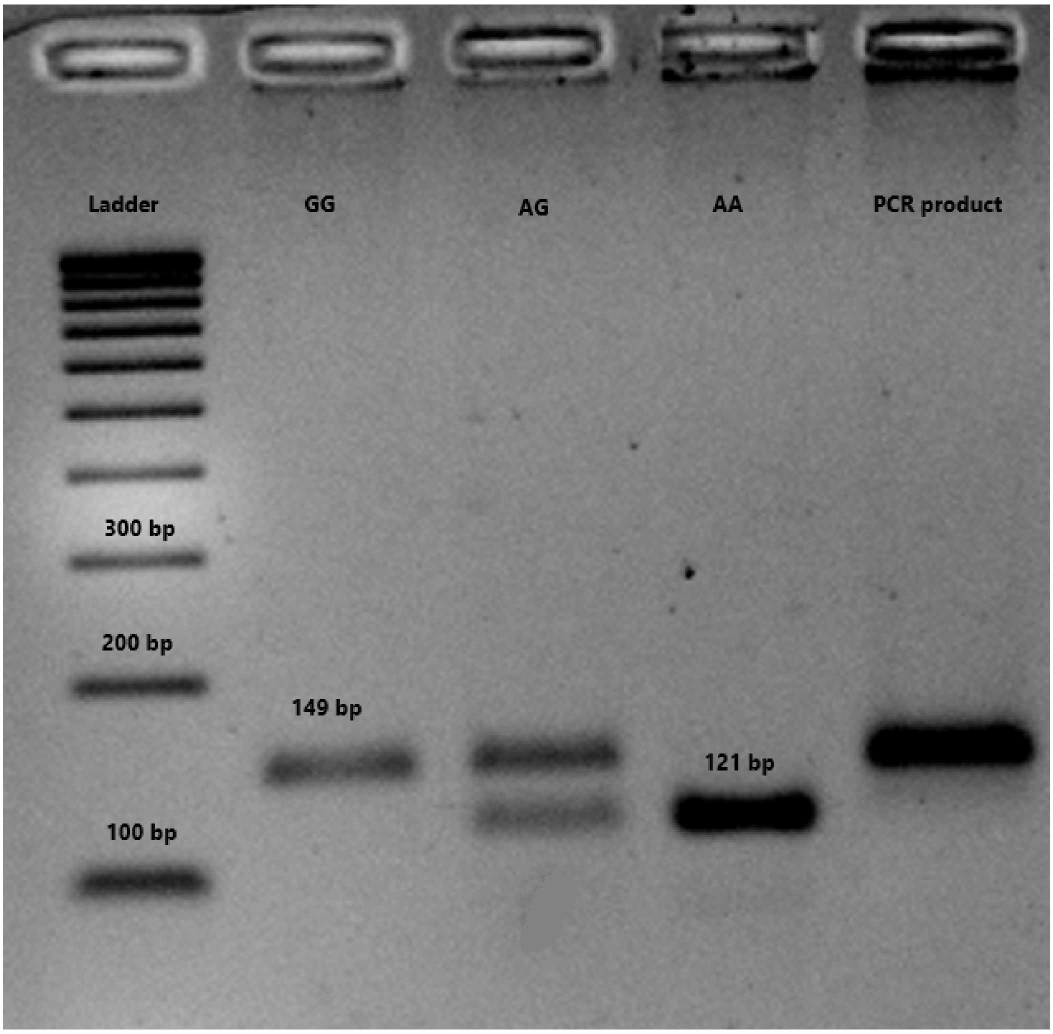

Figure S3 Locus 2q36.3 (rs2943634)

The first lane contains the ladder, the second lane shows the homozygous CC variant (150 bp), the third lane represents the heterozygous AC variant (150/ 124 bp), the fourth lane depicts the homozygous AA variant (124 bp + 26 bp), and the fifth lane displays the non-restricted PCR product (150 bp).

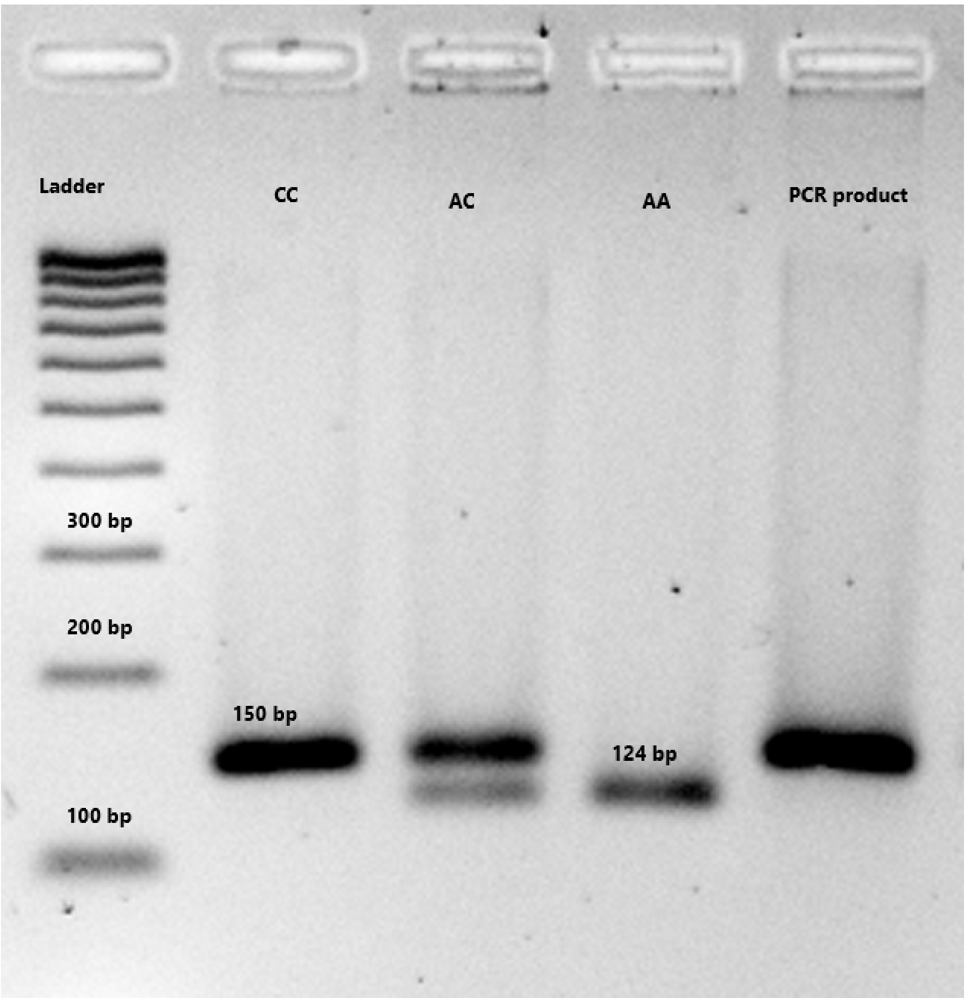

References

Miller SA, Dykes DD, Polesky HF. A simple salting out procedure for extracting DNA from human nucleated cells. Nucleic Acids Res. 1988 Feb 11;16(3):1215. doi: 10.1093/nar/16.3.1215.
